# Low circulating IGF-I bioactivity is associated with human longevity: Findings in centenarians’ offspring

**DOI:** 10.18632/aging.100484

**Published:** 2012-09-09

**Authors:** Giovanni Vitale, Michael P Brugts, Giulia Ogliari, Davide Castaldi, Letizia M. Fatti, Aimee J. Varewijck, Steven W. Lamberts, Daniela Monti, Laura Bucci, Elisa Cevenini, Francesco Cavagnini, Claudio Franceschi, Leo J Hofland, Daniela Mari, Joseph A.M.J.L. Janssen

**Affiliations:** ^1^ Department of Clinical Sciences and Community Health, University of Milan, Milan, Italy; ^2^ IRCCS Istituto Auxologico Italiano, Milan, Italy; ^3^ Department of Internal Medicine, Erasmus Medical Center, Rotterdam, The Netherlands; ^4^ Geriatric Unit IRCCS Ca' Granda Foundation Maggiore Policlinico Hospital, Milan, Italy; ^5^ Dipartimento di Informatica, Sistemistica e Comunicazione, Universita' degli Studi di Milano Bicocca, Milan, Italy; ^6^ Department of Experimental Pathology and Oncology, University of Florence, Florence, Italy; ^7^ C.I.G. Interdepartmental Center “L. Galvani”, University of Bologna, Bologna, Italy; ^8^ Department of Experimental Pathology, University of Bologna, Bologna, Italy

**Keywords:** IGF-I bioactivity, insulin receptors, IGF-I receptors, centenarians’ offspring, centenarians, longevity

## Abstract

Centenarians’ offspring represent a suitable model to study age-dependent variables (e.g. IGF-I) potentially involved in the modulation of the lifespan. The aim of the present study was to investigate the role of the IGF-I in human longevity. We evaluated circulating IGF-I bioactivity measured by an innovative IGF-I Kinase Receptor Activation (KIRA) Assay, total IGF-I, IGFBP-3, total IGF-II, insulin, glucose, HOMA2-B% and HOMA2-S% in 192 centenarians’ offspring and 80 offspring-controls of which both parents died relatively young. Both groups were well-matched for age, gender and BMI with the centenarians’ offspring. IGF-I bioactivity (p<0.01), total IGF-I (p<0.01) and the IGF-I/IGFBP-3 molar ratio (p<0.001) were significantly lower in centenarians’ offspring compared to offspring matched-controls. Serum insulin, glucose, HOMA2-B% and HOMA2-S% values were similar between both groups. In centenarians’ offspring IGF-I bioactivity was inversely associated to insulin sensitivity. In conclusion: 1) centenarians’ offspring had relatively lower circulating IGF-I bioactivity compared to offspring matched-controls; 2) IGF-I bioactivity in centenarians’ offspring was inversely related to insulin sensitivity. These data support a role of the IGF-I/insulin system in the modulation of human aging process.

## INTRODUCTION

Increasing evidence suggests that the insulin-like growth factor-I (IGF-I)/insulin signalling pathway plays a pivotal role in the regulation of longevity [1-6]. In the *Caenorhabditis elegans* and in the *Drosophila melanogaster* it has been suggested that the down-regulation of IGF-I/insulin signalling significantly extends survival [1, 2].

In humans, GH/IGF-I secretion and insulin sensitivity decline with aging [1, 2, 7] and insulin resistance is associated with increased morbidity and mortality [8]. Data on IGF-I system in relation to longevity are still controversial. Bonafè et al. [9] previously found that subjects with at least an A allele of the IGF-I receptor (IGF-IR) gene (G/A, codon 1013) had low levels of free plasma IGF-I and were more represented among long-lived people. In contrast, Paolisso et al. [10] found an increased plasma IGF-I/IGF binding protein 3 (IGFBP-3) molar ratio in healthy centenarians compared to aged subjects. They suggested that this elevated ratio reflected higher IGF-I bioavailability which contributed to the observed improved insulin action in centenarians. An overrepresentation of heterozygous mutations in the IGF-IR gene associated with high serum IGF-I levels and reduced activity of the IGF-IR has been reported in Ashkenazi Jewish centenarians compared to controls [11]. In addition, in humans positive associations between circulating total IGF-I levels and cancer mortality have been found in many studies [12-14], while low total IGF-I levels have been associated with an increased risk for cardiovascular diseases and diabetes [15-22]. On the other hand, Rozing et al. showed that offspring of familial nonagenarians displayed similar IGF-I and IGFBP-3 levels compared to their partners [23].

These conflicting results probably reflect the complexity of the IGF-system. We recently developed an IGF-I kinase receptor activation (KIRA) assay to assess circulating IGF bioactivity [24-27]. This assay determines IGF-I bioactivity by quantifying phosphorylation of tyrosine residues of the activated IGF-IR after stimulation with human serum in vitro. Unlike the traditional IGF-I immunoassays, the IGF-I KIRA assay is able to integrate the modifying effects of IGFBPs and proteases on the interaction between IGF-I and the IGF-IR. Thus by using the IGF-I KIRA it is possible to measure the overall IGF-IR activation in blood [24-27]. Recently it was also found that IGF-I bioactivity, as measured by the IGF-I KIRA, is lower in more insulin sensitive subjects [28], underlining the strict interaction between IGF-I and insulin pathways.

To clarify the role of the IGF-I system in human lifespan, we investigated circulating IGF-I bioactivity and other parameters of the IGF/insulin system in centenarians’ offspring and in a properly selected group of offspring matched-controls. Parameters of the IGF/insulin system were also studied in centenarians and compared with centenarians’ offspring.

## RESULTS

### Health status in centenarians’ offspring and offspring matched-controls

Baseline characteristics of participants are shown in Table 1. Centenarians’ offspring and offspring matched-controls were comparable for age, gender, height, BMI, waist/hip ratio, systolic and diastolic blood pressures, serum total cholesterol and triglycerides levels and smoking prevalence.

**Table 1:** Characteristics of offspring matched-controls, centenarians' offspring and centenarians*

	Offspring matched-controls (n=80)	Centenarians' Offspring (n=192)	Centenarians (n=106)	P^1^	P^2^
**Age (yrs)**	71 (69-75)	71 (65-75)	100 (100-102)	0.66	<0.001
**Male/Females (%)**	34/46 (42.5-57.5)	76/116 (39.6/60.4)	26/80 (24.5/75.5)	0.92	0.03
**Height (cm)**	162.1 ± 8.3	161.9 ± 9	150 ± 8.9	0.94	<0.001
**BMI (kg/m2)**	27.3 (24.8-29.9)	26.1 (23.6-28.7)	23.6 (21.3-26.8)	0.09	<0.001
**Waist/Hip ratio**	0.89 ± 0.08	0.87 ± 0.08	0.89 ± 0.08	0.56	0.17
**Systolic Blood Pressure (mm Hg)**	134 (120-145)	130 (121-140)	125 (120-140)	0.97	<0.01
**Diastolic Blood Pressure (mmHg)**	80 (72-85)	80 (70-82)	70 (60-78)	0.96	<0.001
**Total Cholesterol (mg/dL)**	196 (172-218)	203 (174-231)	185 (163-213)	0.57	0.006
**HDL-Cholesterol (mg/dL)**	56 (46-78)	50 (42-64)	47 (40-55)	0.06	0.01
**Triglycerides (mg/dL)**	105 (79-148)	109 (83-150)	108 (82-143)	0.92	0.97
**Smoking at present: yes/no (%)**	13/66 (16.5/83.5)	23/162 (12.4/87.6)	0/106 (0/100)	0.90	<0.01

* Results are reported as means ± SD for data with a normal distribution, as medians with interquartile ranges (25th-75th percentiles) for data not normally distributed.

1 Offspring matched-controls vs. Centenarians' Offspring

2 Centenarians' Offspring vs. Centenarians

The prevalence of several age-related diseases (Table 2) was lower in centenarians’ offspring than in offspring matched-controls. Differences in prevalence of hypertension (p<0.001) and arrhythmia (p=0.006) were significant between both groups, while differences in prevalence of cancer and osteoarthrosis were borderline significant (both p=0.06). In addition, the proportion of subjects using medications (p=0.001) and the number of prescribed drugs (p<0.001) were significantly lower in centenarians’ offspring than in offspring matched-controls (Table 2).

**Table 2:** Prevalence of some major age-related diseases and medical therapy in offspring matched-controls and centenarians' offspring

Disease/Condition	Offspring matched-controls	Centenarians' Offspring	P-Value^1^
**Myocardial Infarction (%)**	11	6	0.28
**Stroke (%)**	6	2	0.22
**Hypertension (%)**	63	37	<0.001
**Arrhythmia (%)**	23	10	0.006
**Hypercholesterolemia (%)**	47	40	0.18
**Diabetes (%)**	10	10	0.99
**Dementia (%)**	1.3	0.5	0.87
**Cancer (%)**	20	11	0.06
**Osteoporosis (%)**	26	19	0.18
**Osteoarthrosis (%)**	56	44	0.06
***Medical Therapy***			
**Subjects taking drugs (%)**	95	79	0.001
Number of drugs*	4 (2-7)	2 (1-4)	<0.001

1 adjusted for age and gender

* results are reported as medians with interquartile ranges (25th-75th percentiles).

### IGF/insulin system in centenarians’ offspring and offspring matched-controls

Baseline levels of IGF-I system parameters are shown in Table 3. Circulating IGF-I bioactivity, evaluated by KIRA, and total IGF-I were significantly lower in centenarians’ offspring than in offspring matched-controls (both p<0.01). Serum IGFBP-3 (p=0.01) and total IGF-I/IGFBP-3 molar ratio (p<0.001) were respectively higher and lower in centenarians’ offspring than in offspring matched-controls. Differences in IGF-I bioactivity, total IGF-I, IGFBP-3, total IGF-I/IGFBP-3 molar ratio between both groups remained significant after correction for age, gender and BMI (p=0.001, p<0.01, p<0.01 and p<0.001, respectively). No significant differences for IGFBP-2, total IGF-II, glucose, insulin, HOMA2-B% and HOMA2-S% were observed between centenarians’ offspring and offspring matched-controls.

**Table 3:** Parameters of the IGF-I/insulin system in the study population*

	Offspring matched-controls (n=80)	Centenarians' Offspring (n=192)	Centenarians (n=106)	P^1^	P^2^
**IGF-I Bioactivity (pmol/L)**	161 (134-187)	144 (119-170)	132 (107-157)	<0.01	0.09
**Total IGF-I (nmol/L)**	17 (13.6-20.8)	14.4 (11.9-18.2)	9.3 (7.1-12.9)	<0.01	<0.001
**IGFBP-2 (μg/L)**	546 (345-665)	566 (400-678)	728 (603-898)	0.50	<0.001
**IGFBP-3 (nmol/L)**	101.3 (82.7-128.5)	125.8 (107.1-154.5)	79.8 (67.9-92.2)	0.01	<0.001
**Total IGF-I/IGFBP-3 (molar ratio)**	0.15 (0.13-0.17)	0.12 (0.10-0.14)	0.13 (0.09-0.16)	<0.001	0.42
**Total IGF-II (nmol/L)**	114 (89-137)	134 (92-168)	72 (55-117)	0.15	<0.001
**Glucose (mmol/L)**	4.9 (4.5-5.4)	4.8 (4.3-5.4)	4.6 (4.2-5.1)	0.38	0.09
**Insulin (pmol/L)**	74 (51-105)	71 (44-103)	39 (27-70)	0.92	<0.001
**HOMA2-B%**	128 (98-166)	137 (100-174)	109 (81-152)	0.94	<0.01
**HOMA2-S%**	62 (44-93)	68 (45-110)	122 (68-174)	0.90	<0.001

* Results are reported as medians with interquartile ranges (25th-75th percentiles) for data not normally distributed.

1 Offspring matched-controls vs. Centenarians' Offspring

2 Centenarians' Offspring vs. Centenarians

IGF-I system and glucose metabolism interactions in centenarians’ offspring and offspring matched-controls As shown in Table 4, in the centenarians’ offspring group circulating IGF-I bioactivity was positively related to total IGF-I (r=0.26, p=0.001) and negatively to HOMA2-S% (r=-0.18, p<0.05). Similarly, total IGF-I was inversely related to HOMA2-S% (r=-0.18, p<0.05). All these relationships remained significantly after correction for age, gender and BMI, except that between total IGF-I and HOMA2-S% resulting borderline significant (p=0.06).

**Table 4:** Correlation coefficients between parameters of the IGF-I/insulin system in centenarians' offspring and in offspring matched-controls

	IGF-I Bioactivity	Total IGF-I	Total IGF-II	HOMA2-B%
***Centenarians' offspring***				
**Total IGF-I**	0.26^b^			
**Total IGF-II**	0.12	0.12		
**HOMA2-B%**	0.13	0.11	0.14	
**HOMA2-S%**	-0.18^a^	-0.18^a^	-0.09	-0.48^c^
***Offspring matched-controls***				
**Total IGF-I**	0.32^a^			
**Total IGF-II**	0.01	0.05		
**HOMA2-B%**	-0.17	0.03	0.06	
**HOMA2-S%**	0.13	0.24^a^	-0.1	-0.39^c^

a p<0.05

b p=0.001

c p<0.001

In offspring matched-controls (Table 4) circulating IGF-I bioactivity was positively related to total IGF-I (r=0.32, p<0.05). Total IGF-I was also positively related to HOMA2-S% (r=0.24, p<0.05), but after correction for age, gender and BMI, this relationship became borderline significant (p=0.05).

### General characteristics and IGF/insulin system in centenarians

The proportion of women was significantly higher in centenarians than in centenarians’ offspring (Table 1).

Height, BMI, systolic and diastolic blood pressures, total cholesterol levels, HDL levels and smoking prevalence were lower in centenarians compared to their offspring (Table 1). None of the centenarians actually smoked.

Total IGF-I and IGFBP-3 values were significantly lower in centenarians compared to centenarians’ offspring (both p<0.001, Table 3). While there were no significant differences in IGF-I bioactivity and IGF-I/IGFBP-3 molar ratio between both groups. IGFBP-2 and total IGF-II were significantly higher and lower, respectively, in centenarians compared to centenarians’ offspring (both p<0.001, Table 3).

Insulin levels and HOMA2-B% were both significantly lower in centenarians than in the centenarians’ offspring (p<0.001 and p<0.01, respectively, Table 3), while HOMA2-S% was significantly higher in centenarians than in centenarians’ offspring (p<0.001, Table 3).

**Figure 1:**
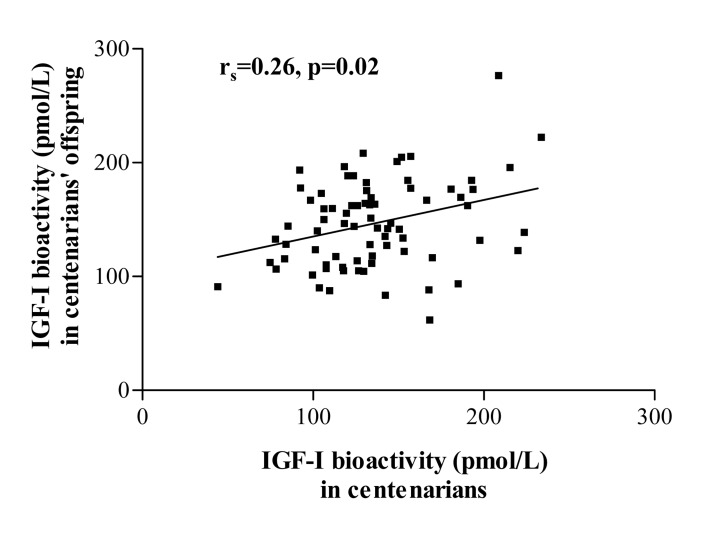
Parent-offspring correlation for IGF-I bioactivity in a subpopulation of 76 centenarians and their corresponding 76 offspring. No such correlation was observed for other IGF-I/insulin system parameters.

In a subpopulation of 76 centenarian parents and their corresponding 76 offspring, IGF-I bioactivity levels showed a significant positive correlation between parent-offspring pairs (r=0.26, p=0.02, Figure 1). No significant associations were found for other IGF/insulin system parameters in parent-offspring pairs.

## DISCUSSION

Although at first glance centenarians seem a good model to study determinants of longevity in humans, there are several limitations of using this approach: low prevalence (1 centenarian per 5-10.000 inhabitants), presence of frailty due to extreme age and lack of a control group of the same age [29, 30]. Due to these restrictions, centenarians are unsuitable to study age-dependent variables (e.g. IGF-I) that may be involved in the modulation of the lifespan. In previous studies circulating IGF-I levels in long-lived people have been often compared to a control group of younger subjects [9, 10]. Therefore, in these studies it was not possible to conclude if IGF-I differences between both groups were expression of a different lifespan or of a physiological age-dependent IGF-I decline.

Centenarians’ offspring are more numerous than centenarians. Several epidemiological studies [31-33] have reported that centenarians’ offspring have a survival advantage and a later onset for several diseases than age-matched controls. Offspring of long-lived siblings were shown to have a more beneficial metabolic profile and a better functional status compared to their controls [34, 35]. In addition, studying centenarians’ offspring has the major advantage of the availability of an appropriate demographically-matched control group (subjects matched for age, sex, ethnicity, parent year of birth, born from non long-lived parents), thereby avoiding cohort effects. Therefore, this strategy seems a logic approach to identify potential factors involved in lifespan and healthy aging.

To the best of our knowledge, this is the first study that evaluated circulating IGF-I bioactivity in centenarians’ offspring. Circulating IGF-I bioactivity, total IGF-I and total IGF-I/IGFBP-3 molar ratio were significantly lower in centenarians’ offspring than in offspring matched-controls and these relationships remained after further adjustment for potential confounders. Both centenarians’ offspring and controls were well-matched for age, gender, height, BMI, waist/hip ratio and smoking habits, while centenarians’ offspring had a significant lower prevalence of some common age-related diseases. In addition, a lower proportion of centenarians’ offspring was treated with drugs. These findings suggest that: 1) the observed differences in circulating IGF-I bioactivity, total IGF-I and IGF-I/IGFBP-3 ratio, were not directly the consequence of differences in age or anthropometric parameters between centenarians’ offspring and offspring matched-controls; 2) centenarians’ offspring were generally more healthy and predisposed to a longer survival than the control group.

Growing evidence indicates a key role of the IGF-I/IGF-IR system in regulating glucose metabolism by influencing β-cell function and insulin sensitivity [36]. IGF-I increases insulin sensitivity not only through its GH-suppressive effects but also by a direct activation of insulin receptor and IGF-IR [37-39]. In the present study, low IGF-I bioactivity in centenarians’ offspring was inversely associated to insulin sensitivity, confirming previous data described in elderly [26] and healthy subjects [28]. This relationship remained after corrections for age, gender and BMI. In addition, centenarians showed a 2-fold higher insulin sensitivity and comparable glucose levels than their younger offspring, despite a lower circulating IGF-I bioactivity, which confirms previous studies reporting that centenarians have lower insulin levels but a better insulin action and glucose tolerance than relatively younger individuals [40, 41].

Caloric restriction has been demonstrated to extend normal life span and improve health in many animal studies [1, 2, 42]. Animals show reduction in IGF-I and insulin levels as well as an increase in the sensitivity to insulin and IGF-I during limited caloric intake [1, 2, 42]. Although only a few studies have really assessed the effects of caloric restriction on lifespan, it has been often suggested that many of the beneficial effects of caloric restriction are attained, at least in part, by down-regulation of the IGF-I receptor/insulin receptor pathways. However, contrary to this widely held idea, it was recently found in an animal model that caloric restriction did not induce an uniform attenuation of IGF-I/insulin signalling [43]. The most surprising finding in this latter study was that caloric restriction induced stimulation of the post-receptor Akt pathway especially in skeletal muscles [43]. An enhanced stimulation of Akt in skeletal muscles of caloric restricted animals is likely to be involved in an improved insulin-mediated glucose disposal. These results provide thus evidence that caloric restriction may improve insulin sensitivity due to a tissue-specific up-regulation of the post-receptor Akt pathway of the insulin receptors and the IGF-I receptors.

**Figure 2:**
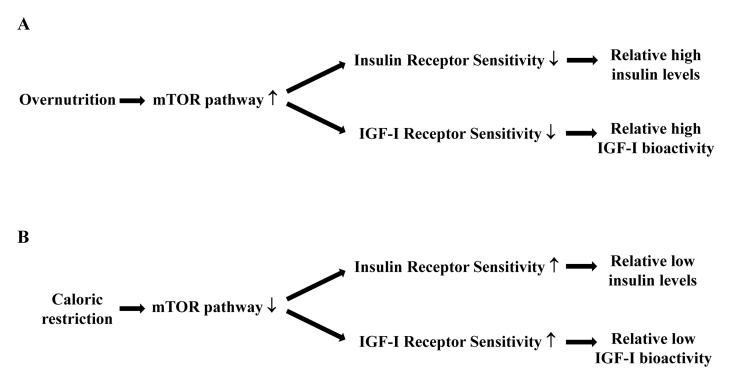
Hypothetical relationships between caloric restriction, the mTOR pathway, insulin and IGF-I receptor sensitivity, circulating insulin levels and IGF-I bioactivity. (**A**) Overnutrition results in an increased activity of the mTOR pathway, which decreases the sensitivity of the insulin and IGF-I receptors. As a consequence there will be relatively high circulating insulin levels and IGF-I bioactivity. (**B**) Caloric restriction results in a reduced activity of the mTOR pathway, which increases the sensitivity of the insulin and IGF-I receptors. As a consequence there will be relatively low circulating insulin levels and IGF-I bioactivity.

A target of rapamycin (mTOR)-centered model may further help to explain why the combination of relatively low IGF-I bioactivity and insulin levels with relatively high insulin responsiveness during caloric restriction is associated with good health and longevity. The mTOR pathway is involved in cellular senescence, aging and age-related diseases [44-46]. Normally the mTOR pathway is activated by numerous signals, including nutrients, IGF-I and insulin [44]. When the mTOR pathway is hyperactivated by overnutrition, it blocks the insulin action on glucose metabolism (Figure 2A). Insulin resistance results in elevated glucose levels and a compensatory insulin increase which will further deteriorate insulin sensitivity. Therefore it has been suggested that overnutrition induces insulin resistance, associated with accelerated aging and a shorter lifespan, through the hyper-activation of the mTOR pathway [44-46]. In contrast, caloric restriction deactivates the mTOR pathway (Figure 2B). This induces an improved insulin sensitivity and relatively low insulin levels and IGF-I bioactivity. Considering that insulin resistance plays a crucial role in determining several age-related diseases, it is reasonable to hypothesize that many of the anti-aging effects of caloric restriction are probably modulated by these pathways [44-46].

Interestingly, several IGF-I/insulin characteristics of the centenarians’ offspring and centenarians in our study are comparable to those found in caloric restricted animals. Although cause and effect may be difficult to assess in a cross-sectional study, we hypothesize that, despite relatively low circulating IGF-I bioactivity, the insulin sensitizing effects of IGF-I at the post-receptor level were also up-regulated in centenarians’ offspring and centenarians.

In favour of our hypothesis, more than 15 years ago Parr [47] suggested that the ideal anti-aging setting for humans would be a high sensitivity to potentially dangerous hormones like IGF-I and insulin. He further suggested that increased IGF-IR and insulin receptor sensitivity, resulting in lower circulating IGF-I and insulin levels, might be the physiological basis for an improved balance of the insulin/IGF-I system. This strategy seems to permit a slower loss of physiologic reserve capacity and a longer lifespan.

Unfortunately, in our study we were not able to measure individual IGF-IR sensitivity, insulin receptor sensitivity and post-receptor pathways of these two receptors to demonstrate that relatively low circulating IGF-I bioactivity in centenarians’ offspring and centenarians is associated with higher IGF-I signalling pathway efficiency at the post-receptor level.

A combination of genetic factors and lifestyle aspects seem to be transmitted to descendants of centenarians, who exhibit better risk profiles compared to age-matched people without centenarians as parents [48]. Interestingly, we found that in contrast to circulating total IGF-I levels, mean IGF-I bioactivity was comparable between centenarians and their offspring. A potential role of the IGF-I system in the familial predisposition of centenarians’ offspring to survive longer is further supported by the observation that only circulating IGF-I bioactivity was significantly correlated in parent-offspring pairs, showing a pattern of familial aggregation. Similarly, Bonafè et al. [9] reported previously that the relatively low levels of immunoreactive free IGF-I found in long-lived subjects were genetically determined and suggested an enhanced tissue response to IGF-I as consequence of an increased IGF-IR number/activity.

In conclusion, we found that: 1) centenarians’ offspring had relatively lower circulating IGF-I bioactivity, but still comparable insulin sensitivity and glucose tolerance than offspring matched-controls; 2) IGF-I bioactivity in centenarians’ offspring was inversely associated to insulin sensitivity; 3) centenarians showed a 2-fold higher insulin sensitivity than centenarians’ offspring. Our data support a role of the IGF-I/insulin system in the modulation of human aging process. We hypothesize that, despite low circulating IGF-I bioactivity, the post-receptor signalling pathways of the IGF-IR were up-regulated in centenarians’ offspring and centenarians. Further studies are needed to confirm this hypothesis.

## METHODS

### Demographic strategy for enrolment and model description

The subjects enrolled belonged to a large multicentric project entitled “Does parental longevity impact on the healthy ageing of their offspring?”. Within this framework, for the present study, we sampled 378 subjects living in Northern and Central Italy (Milan, Bologna and Florence): 106 centenarians (born between 1900-1908) and 192 centenarians’ offspring. Seventy-six out of the 192 centenarians’ offspring were actually offspring of the 106 included centenarians. In addition 80 offspring matched-controls (i.e. subjects age-matched to centenarians’ offspring, but whose parents had died at a relatively young age) were included as controls. In this last group, parents were born in the same birth cohort of centenarians (1900-1908), but they died by natural causes before reaching the average life expectancy calculated by the Italian mortality tables (67 years for males and 72 years for females) (see website “Human Mortality Database” of the Max Plank Institute for Demography, Rostock, Germany: http://www.mortality.org/). The average life expectancy was calculated at 15 years of age. Such an approach takes into account the fact that the parents of the recruited subjects reached by definition the reproductive age. This allows to exclude potential biasing effects of infant mortality.

Subjects were recruited through local advertisements and the Register Office. The participants’ ages were defined by birth certificates or dates of birth as stated on passports or identity cards. The exclusion criteria for the present study were: presence of cancer at the time of the interview, drugs (e.g. insulin, pharmacological doses of corticosteroids, selective estrogen receptor modulators, l-dopa, somatostatin analogues, etc.) and other factors that may actually have influenced IGF-I levels.

A full medical history was obtained from all subjects, followed by a physical examination and recording of systolic and diastolic blood pressure, height, weight, waist and hip circumferences for the assessment of BMI and waist to hip-ratio. A standard structured questionnaire was used to collect information about health status, currently used drugs, clinical history, and life style. This questionnaire was administered by trained staff (nurses and medical doctors). The history of past and current diseases was collected checking the participants’ medical documentation and the current drug therapy. Venous blood samples were drawn between 7.30 and 9.00 a.m. under fasting conditions and immediately processed and remained frozen at −80°C until assayed.

The study protocol was approved by the Ethical Committee of the Sant'Orsola-Malpighi University Hospital (Bologna, Italy) and from all participants written informed consent was obtained.

### Assays

IGF-I bioactivity was measured using an in-house IGF-I KIRA assay, as previously described [24]. Human embryonic renal cells stably transfected with cDNA of the human IGF-IR gene were used as readout after stimulation with either recombinant IGF-I standards or 1/10 diluted serum samples at 37°C. IGF-I standards, two internal control samples, and serum samples from study participants were measured in duplicate on each culture plate. Intraassay and interassay CV were 6.0 and 10.9%, respectively.

Total IGF-I was assayed by one-step sandwich chemiluminescence immunoassay (CLIA) after prior separation of IGF-I from binding proteins on the Liaison autoanalyzer (DiaSorin, Saluggia, Italy). Samples were acidified to separate IGF-I from binding proteins and excess IGF-II was used after acidification in order to avoid residual interference IGF-I with binding proteins. Intraassay and interassay CV were 4.4 and 5.5%, respectively. The mean recovery value was 97% of the hypothetical expected amount.

IGF-II was measured by an Enzyme-Linked Immunosorbent Assay (ELISA), preceded by IGFBPs removal via acid-ethanol extraction, according to the manufacturer's procedure (Diagnostic Systems Laboratories, Webster, Texas). Intraassay and interassay CV were 3.5 and 5.5%, respectively. The mean recovery value was 105% of the hypothetical expected amount.

IGFBP-2 and IGFBP-3 were measured by ELISA according to the manufacturer's procedure (Diagnostic Systems Laboratories, Beckman Coulter, Webster, Texas) in serum samples from 200 out of 378 participants (70 centenarians, 85 centenarians’ offspring and 45 offspring matched controls). Intraassay and interassay CV were 2.5% and 5.1% for IGFBP-2, 8.6% and 9.1% for IGFBP-3, respectively. IGFBP-3 was expressed as nmol/L in order to calculate IGF-I/IGFBP-3 molar ratio, as a surrogate measure for IGF-I bioavailability.

Serum insulin was measured by a CLIA on the fully automated platform Liaison (DiaSorin, Saluggia, Italy). Intraassay and interassay CV were 2.8 and 6%, respectively. Glucose, total cholesterol, HDL-cholesterol and triglycerides were measured by standard laboratory procedures. Fasting indices of pancreatic β-cell function (HOMA2-B%) and insulin sensitivity (HOMA2-S%) were calculated according to the homeostasis model assessment (HOMA) (HOMA Calculator version 2.2.2), available from http://www.dtu.ox.ac.uk [49].

### Statistical analysis

The distribution of continuous variables was tested using the Shapiro-Wilk's test. Data with normal distribution were reported as means and SD, whereas medians and interquartile ranges (25th-75th percentiles) were used for data that did not meet the criteria for normality. For data that did not meet the criteria for normality, natural logarithmic transformations were applied. Comparison between groups was performed by using classical analysis of variance (ANOVA) and Kruskal Wallis test, respectively for parametric and nonparametric data. Bonferroni and Mann-Whitney test (corrected for multiple comparisons) were used as post-tests, respectively for parametric and nonparametric data. Chi-square test was used to analyze categorical variables. Univariate general linear models were used to calculate age, gender and BMI-adjusted differences in the mean values of variables between groups. F test was used to calculate significance. Correlations between insulin/IGF-I system parameters were presented as Spearman correlation coefficients and partial correlation coefficients adjusted for potential confounding variables. All analyses were carried out using SPSS for Windows, release 17.0. A two-tailed p-value of < 0.05 was considered significant.

## References

[R1] Longo VD, Finch CE (2003). Evolutionary medicine: from dwarf model systems to healthy centenarians?. Science.

[R2] Fontana L, Partridge L, Longo VD (2010). Extending healthy life span-from yeast to humans. Science.

[R3] Franceschi C, Olivieri F, Marchegiani F, Cardelli M, Cavallone L, Capri M, Salvioli S, Valensin S, De Benedictis G, Di Iorio A, Caruso C, Paolisso G, Monti D (2005). Genes involved in immune response/inflammation, IGF1/insulin pathway and response to oxidative stress play a major role in the genetics of human longevity: the lesson of centenarians. Mech Ageing Dev.

[R4] Ugalde AP, Mariño G, López-Otín C (2010). Rejuvenating somatotropic signaling: a therapeutical opportunity for premature aging?. Aging (Albany NY).

[R5] Belfiore A, Frasca F, Pandini G, Sciacca L, Vigneri R (2009). Insulin receptor isoforms and insulin receptor/insulin-like growth factor receptor hybrids in physiology and disease. Endocr Rev.

[R6] Van Heemst D, Beekman M, Mooijaart SP, Heijmans BT, Brandt BW, Zwaan BJ, Slagboom PE, Westendorp RG (2005). Reduced insulin/IGF-1 signalling and human longevity. Aging Cell.

[R7] Aimaretti G, Boschetti M, Corneli G, Gasco V, Valle D, Borsotti M, Rossi A, Barreca A, Fazzuoli L, Ferone D, Ghigo E, Minuto F (2008). Normal age-dependent values of serum insulin growth factor-I: results from a healthy Italian population. J Endocrinol Invest.

[R8] Kuk JL, Saunders TJ, Davidson LE, Ross R (2009). Age-related changes in total and regional fat distribution. Ageing Res Rev.

[R9] Bonafe M, Barbieri M, Marchegiani F, Olivieri F, Ragno E, Giampieri C, Mugianesi E, Centurelli M, Franceschi C, Paolisso G (2003). Polymorphic variants of insulin-like growth factor I (IGF-I) receptor and phosphoinositide 3-kinase genes affect IGF-I plasma levels and human longevity: cues for an evolutionarily conserved mechanism of lifespan control. J Clin Endocrinol Metab.

[R10] Paolisso G, Ammendola S, Del Buono A, Gambardella A, Riondino M, Tagliamonte MR, Rizzo MR, Carella C, Varricchio M (1997). Serum levels of IGF-I and IGFBP-3 in healthy centenarians. J Clin Endocrinol Metab.

[R11] Suh Y, Atzmon G, Cho MO, Hwang D, Liu B, Leahy DJ, Barzilai N, Cohen P (2008). Functionally significant insulin-like growth factor I receptor mutations in centenarians. Proc Natl Acad Sci USA.

[R12] Renehan AG, Zwahlen M, Minder C, O'Dwyer ST, Shalet SM, Egger M (2004). Insulin-like growth factor (IGF)-I, IGF binding protein-3, and cancer risk: systematic review and meta-regression analysis. Lancet.

[R13] Rowlands MA, Gunnell D, Harris R, Vatten LJ, Holly JM, Martin RM (2009). Circulating insulin-like growth factor peptides and prostate cancer risk: a systematic review and meta-analysis. Int J Cancer.

[R14] Major JM, Laughlin GA, Kritz-Silverstein D, Wingard DL, Barrett-Connor E (2010). Insulin-Like Growth Factor-I and Cancer Mortality in Older Men. J Clin Endocrinol Metab.

[R15] Janssen JA, Lamberts SW (2004). Igf-I and longevity. Horm Res.

[R16] Janssen JA, Stolk RP, Pols HA, Grobbee DE, Lamberts SW (1998). Serum total IGF-I, free IGF-I, and IGFBP-1 levels in an elderly population: relation to cardiovascular risk factors and disease. Arterioscler Thromb Vasc Biol.

[R17] Johnsen SP, Hundborg HH, Sørensen HT, Orskov H, Tjønneland A, Overvad K, Jørgensen JO (2005). Insulin-like growth factor (IGF) I, -II, and IGF binding protein-3 and risk of ischemic stroke. J Clin Endocrinol Metab.

[R18] Juul A, Scheike T, Davidsen M, Gyllenborg J, Jørgensen T (2002). Low serum insulin-like growth factor I is associated with increased risk of ischemic heart disease: a population-based case-control study. Circulation.

[R19] Colao A, Di Somma C, Cascella T, Pivonello R, Vitale G, Grasso L, Lombardi G, Savastano S (2008). Relationships between serum insulin-like growth factor-I levels, blood pressure and glucose tolerance: an observational, exploratory study in 404 subjects. Eur J Endocrinol.

[R20] Colao A, Di Somma C, Filippella MG, Rota F, Pivonello R, Orio F, Vitale G, Lombardi G (2004). Insulin-like growth factor deficiency determines increased intima-media thickness at common carotid arteries in adult patients with growth hormone deficiency. Clin Endocrinol.

[R21] Galderisi M, Vitale G, Lupoli G, Barbieri M, Varricchio G, Carella C, de Divitiis O, Paolisso G (2001). Inverse association between free insulin-like growth factor-1 and isovolumic relaxation in arterial systemic hypertension. Hypertension.

[R22] Galderisi M, Caso P, Cicala S, De Simone L, Barbieri M, Vitale G, de Divitiis O, Paolisso G (2002). Positive association between circulating free IGF-1 levels and coronary flow reserve in arterial systemic hypertension. Am J Hypertens.

[R23] Rozing MP, Westendorp RG, Frölich M, de Craen AJ, Beekman M, Heijmans BT, Mooijaart SP, Blauw GJ, Slagboom PE, van Heemst D, Leiden Longevity Study (LLS) Group (2009). Human insulin/IGF-1 and familial longevity at middle age. Aging (Albany NY).

[R24] Brugts MP, Ranke MB, Hofland LJ, van der Wansem K, Weber K, Frystyk J, Lamberts SW, Janssen JA (2008). Normal values of circulating insulin-like growth factor-I bioactivity in the healthy population: comparison with five widely used IGF-I immunoassays. J Clin Endocrinol Metab.

[R25] Brugts MP, van den Beld AW, Hofland LJ, van der Wansem K, van Koetsveld PM, Frystyk J, Lamberts SW, Janssen JA (2008). Low circulating insulin-like growth factor I bioactivity in elderly men is associated with increased mortality. J Clin Endocrinol Metab.

[R26] Brugts MP, van Duijn CM, Hofland LJ, Witteman JC, Lamberts SW, Janssen JA (2010). Igf-I bioactivity in an elderly population: relation to insulin sensitivity, insulin levels, and the metabolic syndrome. Diabetes.

[R27] Chen JW, Ledet T, Orskov H, Jessen N, Lund S, Whittaker J, De Meyts P, Larsen MB, Christiansen JS, Frystyk J (2003). A highly sensitive and specific assay for determination of IGF-I bioactivity in human serum. Am J Physiol Endocrinol Metab.

[R28] Arafat AM, Weickert MO, Frystyk J, Spranger J, Schöfl C, Möhlig M, Pfeiffer AF (2009). The role of insulin-like growth factor (IGF) binding protein-2 in the insulin-mediated decrease in IGF-I bioactivity. J Clin Endocrinol Metab.

[R29] Franceschi C, Bonafè M (2003). Centenarians as a model for healthy aging. Biochem Soc Trans.

[R30] Cevenini E, Invidia L, Lescai F, Salvioli S, Tieri P, Castellani G, Franceschi C (2008). Human models of aging and longevity. Expert Opin Biol Ther.

[R31] Terry DF, Wilcox MA, McCormick MA, Pennington JY, Schoenhofen EA, Andersen SL, Perls TT (2004). Lower all-cause, cardiovascular, and cancer mortality in centenarians’ offspring. J Am Geriatr Soc.

[R32] Adams ER, Nolan VG, Andersen SL, Perls TT, Terry DF (2008). Centenarian offspring: Start healthier and stay healthier. J Am Geriatr Soc.

[R33] Atzmon G, Schechter C, Greiner W, Davidson D, Rennert G, Barzilai N (2004). Clinical Phenotype of Families with Longevity. J Am Geriatr Soc.

[R34] Mooijaart SP, van Heemst D, Noordam R, Rozing MP, Wijsman CA, de Craen AJ, Westendorp RG, Beekman M, Slagboom PE (2011). Polymorphisms associated with type 2 diabetes in familial longevity: The Leiden Longevity Study. Aging (Albany NY).

[R35] Newman AB, Glynn NW, Taylor CA, Sebastiani P, Perls TT, Mayeux R, Christensen K, Zmuda JM, Barral S, Lee JH, Simonsick EM, Walston JD, Yashin AI, Hadley E (2011). Health and function of participants in the Long Life Family Study: A comparison with other cohorts. Aging (Albany NY).

[R36] Clemmons DR (2012). Metabolic actions of insulin-like growth factor-I in normal physiology and diabetes. Endocrinol Metab Clin North Am.

[R37] Mohn A, Marcovecchio ML, de Giorgis T, Pfaeffle R, Chiarelli F, Kiess W (2011). An insulin-like growth factor-I receptor defect associated with short stature and impaired carbohydrate homeostasis in an Italian pedigree. Horm Res Paediatr.

[R38] O'Connell T, Clemmons DR (2002). IGF-I/IGF-binding protein-3 combination improves insulin resistance by GH-dependent and independent mechanisms. J Clin Endocrinol Metab.

[R39] Rajpathak SN, Gunter MJ, Wylie-Rosett J, Ho GY, Kaplan RC, Muzumdar R, Rohan TE, Strickler HD (2009). The role of insulin-like growth factor-I and its binding proteins in glucose homeostasis and type 2 diabetes. Diabetes Metab Res Rev.

[R40] Paolisso G, Gambardella A, Ammendola S, D'Amore A, Balbi V, Varricchio M, D'Onofrio F (1996). Glucose tolerance and insulin action in healty centenarians. Am J Physiol.

[R41] Barbieri M, Rizzo MR, Manzella D, Paolisso G (2001). Age-related insulin resistance: is it an obligatory finding? The lesson from healthy centenarians. Diabetes Metab Res Rev.

[R42] D'Costa AP, Lenham JE, Ingram RL, Sonntag WE (1993). Moderate caloric restriction increases type 1 IGF receptors and protein synthesis in aging rats. Mech Ageing Dev.

[R43] Sharma N, Castorena CM, Cartee GD (2012). Tissue-Specific Responses of IGF-1/Insulin and mTOR Signaling in Calorie Restricted Rats. PLoS One.

[R44] Blagosklonny MV (2010). Calorie restriction: decelerating mTOR-driven aging from cells to organisms (including humans). Cell Cycle.

[R45] Blagosklonny MV (2010). Revisiting the antagonistic pleiotropy theory of aging: TOR-driven program and quasi-program. Cell Cycle.

[R46] Blagosklonny MV (2012). Once again on rapamycin-induced insulin resistance and longevity: despite of or owing to. Aging (Albany NY).

[R47] Parr T (1996). Insulin exposure controls the rate of mammalian aging. Mech Ageing Dev.

[R48] Galioto A, Dominguez LJ, Pineo A, Ferlisi A, Putignano E, Belvedere M, Costanza G, Barbagallo M (2008). Cardiovascular risk factors in centenarians. Exp Gerontol.

[R49] Wallace TM, Levy JC, Matthews DR (2004). Use and abuse of HOMA modeling. Diabetes Care.

